# Measurement error, time lag, unmeasured confounding: Considerations for longitudinal estimation of the effect of a mediator in randomised clinical trials

**DOI:** 10.1177/0962280216666111

**Published:** 2016-09-19

**Authors:** KA Goldsmith, T Chalder, PD White, M Sharpe, A Pickles

**Affiliations:** 1 Biostatistics & Health Informatics Department, Institute of Psychiatry, Psychology & Neuroscience, King’s College London, London, UK; 2 Academic Department of Psychological Medicine, Weston Education Centre, Institute of Psychiatry, Psychology & Neuroscience, King’s College London, London, UK; 3 Centre for Psychiatry, Wolfson Institute of Preventive Medicine, Barts and the London School of Medicine, Queen Mary University, London, UK; 4 Psychological Medicine Research, Department of Psychiatry, University of Oxford, Oxford, UK

**Keywords:** Mediation, longitudinal mediation models, structural equation models, measurement error, clinical trials, chronic fatigue syndrome

## Abstract

Clinical trials are expensive and time-consuming and so should also be used to study how treatments work, allowing for the evaluation of theoretical treatment models and refinement and improvement of treatments. These treatment processes can be studied using mediation analysis. Randomised treatment makes some of the assumptions of mediation models plausible, but the mediator–outcome relationship could remain subject to bias. In addition, mediation is assumed to be a temporally ordered longitudinal process, but estimation in most mediation studies to date has been cross-sectional and unable to explore this assumption. This study used longitudinal structural equation modelling of mediator and outcome measurements from the PACE trial of rehabilitative treatments for chronic fatigue syndrome (ISRCTN 54285094) to address these issues. In particular, autoregressive and simplex models were used to study measurement error in the mediator, different time lags in the mediator–outcome relationship, unmeasured confounding of the mediator and outcome, and the assumption of a constant mediator–outcome relationship over time. Results showed that allowing for measurement error and unmeasured confounding were important. Contemporaneous rather than lagged mediator–outcome effects were more consistent with the data, possibly due to the wide spacing of measurements. Assuming a constant mediator–outcome relationship over time increased precision.

## 1 Introduction

The primary objective of randomised clinical trials is to answer the question ‘does the treatment work’? However, researchers generally have other important questions about treatment, such as how treatments work, and the testing of these theoretical models of treatment mechanisms provides essential knowledge for future therapeutic development. The case is especially strong when a trial shows no treatment differences, when it is important to obtain information about why the treatment failed. Trials are expensive and can be demanding for both patients and clinical staff. Answering questions beyond those of treatment effectiveness can be justified in terms of marginal cost, burden and ethics, and in many instances may require little more than additional analyses. Such investigations often rely heavily on mediation analysis. While mediation analysis is in very common use, with one of the source works by Baron and Kenny having been cited more than 10,000 times, studies applying these methods rarely discuss the problems of variables measured with error and post-randomisation confounding. This paper describes how these problems can be addressed in a structural equation modelling framework.

**Figure 1.**
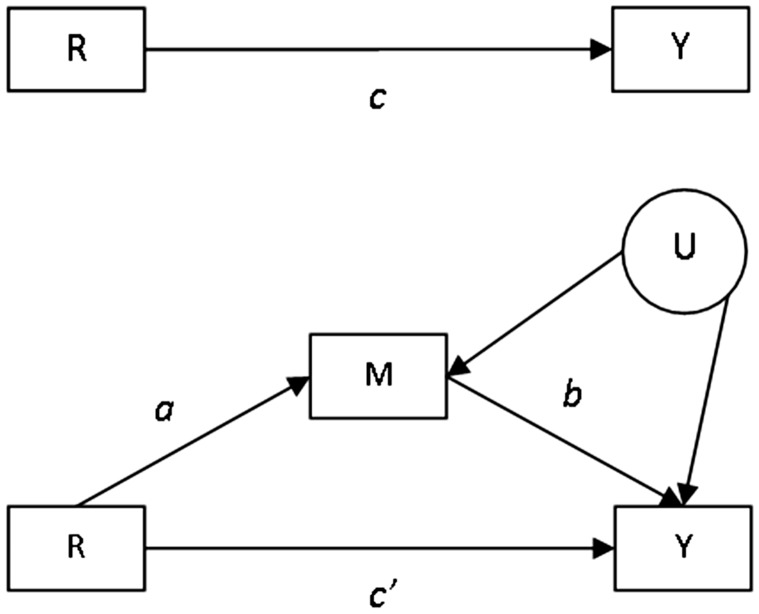
Treatment effect and mediation path diagrams. R: randomised treatment; Y: outcome; M: mediator; U: unmeasured confounders.

A mediation model is depicted at the bottom of Figure 1, each path labelled as is traditional for this field. Under certain no unmeasured confounding assumptions, correct model specification and when the mediator and outcome are continuous, mediation can be assessed through a series of ordinary least squares regression equations where Y = outcome, M = mediator and R = randomly assigned treatment^1^:

(1) $$Y_{i}=\alpha_{1}+\beta_{1}R_{i}+{ɛ}_{i1}$$

(2) $$M_{i}=\alpha_{2}+\beta_{2}R_{i}+{ɛ}_{i2}$$

(3) $$Y_{i}=\alpha_{3}+\beta_{3}R_{i}+\gamma M_{i}+{ɛ}_{i3}$$

The *i* subscript refers to the unit (often the individual) receiving treatment. The *α*_1_, *α*_2_ and *α*_3_ parameters are intercepts, with *ɛ*_i1_, *ɛ*_i2_, *ɛ*_i3_ representing the error terms in the regression equations. *β*_1_ gives the total effect of treatment on outcome, referred to in the mediation literature as the *c* path. In the past, it was suggested that mediation should only be assessed when this total effect was significant, but more recently we and others have argued that mediation should be assessed whether or not β_1_ is significant.^2–7^ In order for there to be mediation, β_2_ in equation (2) (*a* in Figure 1), should be significant; i.e. treatment has an effect on the mediator. In addition, in equation (3), where γ corresponds to *b* in Figure 1, γ should be significant, i.e. the mediator has an effect on the outcome. The indirect, or mediated, effect is then given by β_2_γ (or the product *a* times *b*), known as the product of coefficients (POC) estimate.^8^ Any remaining direct effect of treatment on the outcome is given by β_3_, which is referred to as *c’*. In the situation where β_1_ is not significant, studying these relationships could clarify why the treatment was not effective. The estimated *a* path shows whether the treatment had any effect on the mediator, while the estimated *b* path clarifies whether the mediator is predictive of the outcome. Non-significance of either of these estimates could partly explain the ineffectiveness of a treatment. Studying the *a*, *b* and *c’* paths together will also indicate whether the direction of the *c’* path or direct effect is different from that of the indirect effect. This is referred to as inconsistent mediation or suppression and can be associated with a non-significant overall effect.^3^ The POC mediated effect estimate is a simple example of the application of Sewell Wright’s path tracing rules, which takes the products of the estimates along each legal path between two variables and then sums the products across all of the paths.^9,10^ Paths cannot be traced: (1) where an arrow is first traced in a forward direction and the next arrow in a backwards direction, (2) through a variable more than once and (3) through more than one bidirectional arrow. It is important to note that this tracing and indirect effect calculation assumes linear relationships amongst the variables in the model and conditional multivariate normality.^11,12^ The models in (2) and (3) can be fitted simultaneously using the structural equation model (SEM) framework. We focus here on how the framework allows some progress to be made in relation to two of the likely major sources of bias in mediation effect estimates, namely measurement error in the mediator and confounding of the mediator–outcome relationship – the potential for the latter is shown as U in Figure 1.

Classical measurement error in the mediator would result in a bias towards zero in the estimate of the mediator, so-called attenuation or dilution.^13^ The single mediator/single outcome model of Figure 1 can account for measurement error if a good estimate of the reliability of the mediator is known,^14^ and other regression-based methods have also recently been applied to address this problem.^15,16^ However, SEM allows for the simultaneous estimation of multiple equations and with repeated or multivariate measurement this allows the incorporation of measurement error using latent variables.^11,12,17^ The latent or ‘true score’ is a hypothetical quantity relating to the error-free measure of the construct of interest, which is defined in practice from a decomposition of covariances.^11,12^ In SEM with repeated measures this commonly follows a classical measurement error model, in which the variance of a measure is partitioned into a latent ‘true score’ and an additive error which is assumed conditionally independent.^11–13^ The error is also assumed to be independent of this true latent mediator score given exposure and covariates. The effect of a latent mediator variable on the outcome would provide an estimate of the mediator–outcome relationship disattenuated for measurement error.^11,12^

In randomised clinical trials, the mediator is not generally randomised, and as a consequence, the relationship between the mediator and outcome could be subject to confounding, which we have shown as U in Figure 1.^3,18–21^ The need to make the assumption of no unmeasured confounding has received much attention in the literature.^2,3,22–30^ Extending the range of measured and included confounders is recommended, but without the addition of an instrumental variable, which in practice is rarely available,^4,14^ we cannot add an error covariance between mediator and outcome to the simple model of Figure 1 as the model will no longer be identified. This is because there would be six parameters to estimate (three regression parameters, two error variances and an error covariance) and only five quantities available for estimation (two variances and three covariances). Quantifying parameter estimate sensitivity to unmeasured confounding provides another option. For example, VanderWeele describes general direct and indirect effects bias formulae for sensitivity analysis,^27^ while Imai and colleagues use the SEM framework as a starting point, and provide methods to quantify the sensitivity of the mediator–outcome relationship to the correlation between mediator and outcome error terms.^23,24^ We highlight that with repeated measurement, relaxation of the conditional independence assumption of SEM measurement models is possible and can allow for correlated measurement errors that some unmeasured confounders may induce between the mediator and outcome. This has allowed us to take a more model selection-based approach using model fit criteria and perusal of the effect of different SEM on the mediator–outcome relationship estimates. Fitting different SEM in this way could be thought of another type of sensitivity analysis. This method has some advantages over other sensitivity analysis solutions that have been offered,^15,16,23,24^ in that it allows for measurement error and unmeasured confounding in a single step, and unlike regression based methods, does not require the provision of sensitivity parameters such as measurement reliability and measurement error variance.^15,16^ Such parameters are likely to be difficult to estimate accurately. De Stavola and colleagues extended the sensitivity analyses described by Imai et al. to the situation where there are confounders of the mediator and outcome that are affected by the treatment or ‘intermediate confounders’.^22,25,26,28,29^ We make the assumption for the purposes of this paper that we have no ‘intermediate confounding’.

The study that motivated this work was the **P**acing, Graded **A**ctivity, and **C**ognitive Behaviour Therapy: A Randomised **E**valuation or PACE study of rehabilitative treatments for Chronic Fatigue Syndrome (CFS),^31^ which is described in more detail in the Methods section. In an initial study of mediation of the PACE treatments using the POC method,^3,8^ the mid-treatment measurement of the mediators and the follow-up measure of outcomes showed that the effects of cognitive behavioural therapy and graded exercise therapy were partially mediated through cognitive and behavioural factors.^32^ However, this initial analysis did not take advantage of all the available repeated measures data. Mediation is regarded as a causal process, where the temporal ordering of treatment->mediator->outcome is implied. The repeated measures of mediator and outcome data in the PACE trial allow for exploration of time lags in mediated effects in addition to measurement error and unmeasured confounding.

The aim of this work was to address issues affecting estimation and precision of the mediator–outcome relationship, or *b* path, in longitudinal mediation models. These issues were: measurement error in the mediator, time lags of mediator–outcome relationship, unmeasured confounding and the assumption of a constant mediator–outcome relationship over time. The repeated measures of mediators and outcomes available in the PACE data were used to address this aim.

## 2 Methods

### 2.1 Motivating study

The PACE trial of treatments for CFS randomised individuals to four treatment groups, which were three different therapies each in addition to the control treatment, and the control treatment alone.^31^ The therapies were cognitive behavioural therapy (CBT), graded exercise therapy (GET) and adaptive pacing therapy (APT), with the control being specialist medical care (SMC) delivered by a doctor with experience of treating CFS. CBT was delivered by psychotherapists and focused on examining the link between thoughts, behaviours and symptoms and negotiating behavioural and cognitive changes with the patient as appropriate. It was based on a fear avoidance model of CFS.^32^ GET was delivered by physiotherapists and involved a mutually agreed gradual increase in activity for the patient. It was based on deconditioning and exercise intolerance models of the illness. APT was delivered by occupational therapists. It was based on a model of finite and limited amounts of energy and advised participants to balance activity with rest. The trial found that CBT and GET were superior to APT and SMC for both physical functioning and fatigue outcomes. Measures of the mediators and outcomes were taken at baseline, mid-treatment (12 weeks post-randomisation), post-treatment (24 weeks post-randomisation) and follow-up (52 weeks post-randomisation). The West Midlands Multicentre Research Ethics Committee (MREC 02/7/89) approved the original PACE study, ISRCTN 54285094. This trial provides a unique opportunity to study longitudinal mediation given that most mediators were measured at all time points, the mediators and outcomes were measured mid-therapy, which is uncommon, and there were four treatment groups rather than the usual two treatment group design. The data on the subset of the participants randomised to receive CBT and APT were used for this analysis shown here. Advantages associated with the four treatment group design and longitudinal mediation results for all four treatment groups will be reported elsewhere.

An example mediator, fear avoidance beliefs, and one of the two primary trial outcomes, self-rated physical functioning, were used in this study. Fear avoidance beliefs were measured using the Cognitive Behavioural Responses Questionnaire,^33,34^ which is a scale ranging from 0 to 24. Physical functioning was measured with the physical functioning subscale of the SF-36,^35,36^ which ranges from 0 to 100. Fear avoidance beliefs were found to be the strongest mediator of the effect of CBT and GET as compared to APT in the simple single mediator/outcome POC analysis.^32^

### 2.2 Structural equation models

Data were standardised using the mean and standard deviation (SD) of the baseline measure, so the units for the mediator–outcome estimates and indirect effects were baseline physical functioning SDs. SEMs were used to model the longitudinal mediation and outcome processes. The only other covariate in the models was treatment group (CBT = 1 versus APT = 0). Mplus version 7.2 was used to fit models; full information maximum likelihood was used, which estimates parameters using information from complete and incomplete records under a missing at random assumption.^37,38^ Model fit was assessed using the chi-square test of model fit^12^ and the root mean square error of approximation (RMSEA).^39,40^ Models were informally compared using the Chi-square statistic, RMSEA and the Bayesian Information Criterion (BIC).^41^

### 2.3 Longitudinal structural equation models for mediation

**Figure 2.**
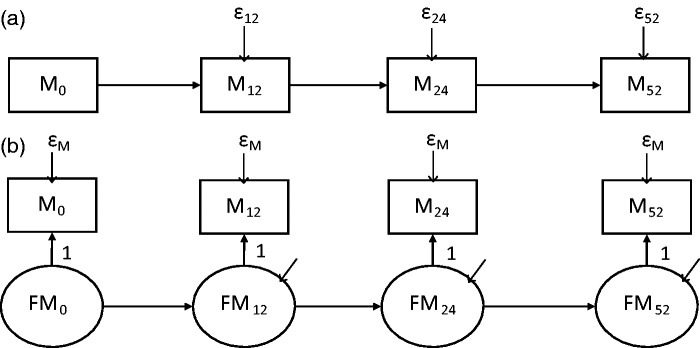
Autoregressive and simplex model examples for the mediator process. M_0_: mediator at baseline; M_12_: mediator at 12 weeks; M_24_: mediator at 24 weeks; M_52_: mediator at 52 weeks; FM_0_: latent true mediator score at baseline; FM_12_: latent true mediator score at 12 weeks; FM_24_: latent true mediator score at 24 weeks; FM_52_: latent true mediator score at 52 weeks. ɛ_12_, ɛ_24_ and ɛ_52_ are the mediator error variances at 12, 24 and 52 weeks. One method for obtaining identification of the model in (b) is shown in the figure: all the factor loadings = 1 and var(ɛ) are set equal.

**Figure 3.**
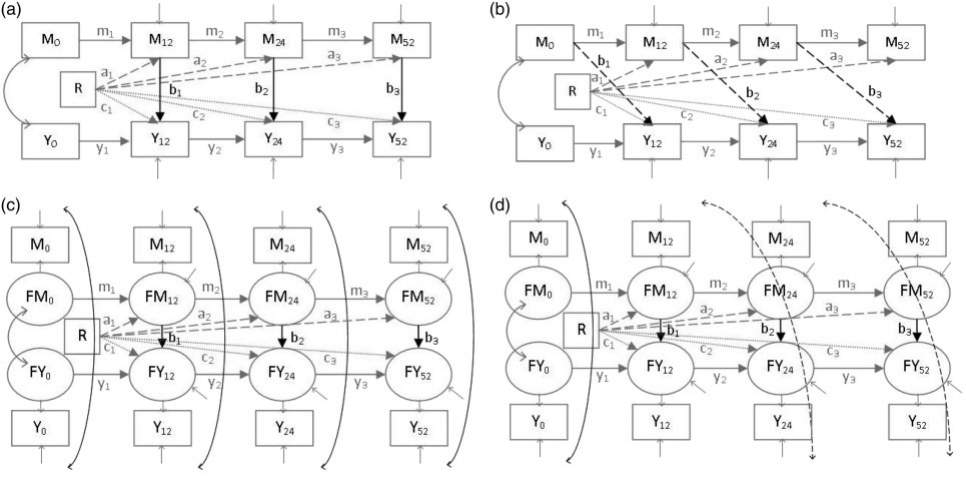
Autoregressive models with contemporaneous and lagged *b* paths and simplex models with contemporaneous and lagged covariances using PACE example time points. M_0_, M_12_, M_24_, M_52_: mediator measurements taken at baseline, 12 weeks, 24 weeks and 52 weeks post-randomisation. Y_0_, Y_12_, Y_24_, Y_52_: outcome measurements taken at the same time points. FM_0_: latent true mediator score at baseline, FM_12_: latent true mediator score at 12 weeks, FM_24_: latent true mediator score at 24 weeks, FM_52_ = latent true mediator score at 52 weeks, FY_0_, FY_12_, FY_24_, FY_52_ = latent true outcome scores as described for the mediator.

There are several longitudinal SEMs that could be applied in a mediation analysis, which have been reviewed by MacKinnon.^3^ One such model is a first-order autoregressive as shown in Figure 2(a) for a mediator process where measures have been taken at the time points in the PACE trial. The model assumes that: each variable is a function only of the measure of that variable at the previous time point (plus other covariates such as treatment group), there is no measurement error and correlations between the measurements decrease over time.^3,42,43^ This may or may not be a plausible assumption for the correlations of the mediator and outcome measurements in PACE and other clinical trials. When using such models for mediation, an autoregressive structure is fit to both the mediator and outcome processes separately and then these are linked together by estimates we will refer to as the *b* paths,^3^ as shown in Figure 3. Figure 3 also shows labelling of the *a* and *c*' paths in the longitudinal models; we have labelled and will refer to the *c*' paths as *c* paths for simplicity. The exposure of interest, here R or in other words the randomised treatment group, is allowed to affect various measures of both the mediator and outcome over time.

We explored the effects of the following on estimation and precision of the *b*_1_, *b*_2_ and *b*_3_ paths (Figure 3) within the autoregressive model structure:

1. allowing for independent measurement error in the mediators to remove attenuation bias,
2. allowing different *b*_1_, *b*_2_ and *b*_3_ path time lags,
3. allowing for measurement error covariances between the mediator and outcome variables that would be induced by unmeasured confounding and which could attenuate or inflate the *b*_1_, *b*_2_ and *b*_3_ path estimates and
4. assuming equality of the post-randomisation *b* paths over time, which would be consistent with the view that no matter how or when the mediator is changed, its relationship with the outcome remains constant.

Before reporting results we detail how each of these issues were addressed in the modelling. All the models leave the randomised treatment group to be uncorrelated with all other variables.

### 2.4 Measurement error – Autoregressive versus simplex models

**Figure 4.**
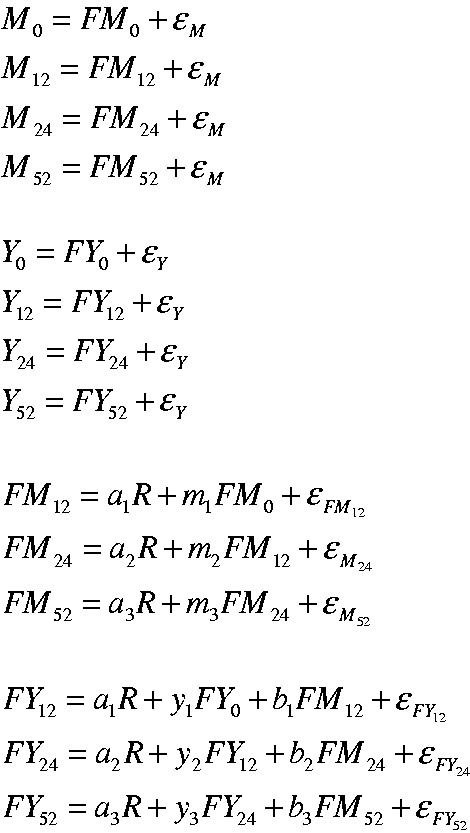
Equations describing dual-process simplex model shown in Figure 3(c). Models were fitted with mediator (M) error variances set equal (ɛ_M_) and outcome (Y) error variances set equal (ɛ_Y_).

Autoregressive models can be extended to allow for measurement error by fitting models with a quasi-simplex structure as shown in Figure 2(b) for the mediator process. The quasi-simplex model (referred to as simplex hereafter) uses each observed value of the variable as a single indicator for a latent ‘true score’ variable with the autoregressive relationship for the variable then being between these true scores rather than the observed scores.^42–44^ In this way, classical measurement error is incorporated.^11–13^ So if for example we take the baseline mediator measure, M_0_, it is made up of the true measure, FM_0_, plus the conditionally independent error, ${ɛ}_{{\text{M}}_{0}}$ or ${\text{M}}_{0}={\text{FM}}_{0}+{ɛ}_{{\text{M}}_{0}}$. One way to obtain identification of a simplex model with four measurements is to assume parallel measurement and set the factor loadings all equal to one and the error variances equal over time. This was the approach taken here. The importance of allowing for measurement error was assessed by using the dual-process autoregressive and simplex models fitted to both the mediator and outcome processes as shown in Figure 3 and comparing model fit, as well as informally comparing the *b* path estimates and their standard errors (i.e. without using statistical tests). Figure 4 shows the equations associated with the model in Figures 3(c), assuming classical measurement error.^13^

### 2.5 Time lags of *b* paths – Lagged and contemporaneous

In each case, an autoregressive or simplex model was fitted with either lagged *b*_1_, *b*_2_ and *b*_3_ paths between the mediator at the time point prior to the outcome at the time point immediately following, or contemporaneous *b*_1_, *b*_2_ and *b*_3_ paths between mediator and outcome at the same time point. Lagged paths respect the implied temporal ordering of change in mediator prior to change in outcome that would be expected for a causal process. Models with contemporaneous paths were also fitted despite their lack of allowance for temporal ordering as these sorts of relations were plausible, perhaps especially given the considerable lengths of time between measures. Autoregressive models with these two types of paths are shown in Figures 3(a) and (b). Figures 3(c) and (d) show dual process simplex models with contemporaneous *b*_1_, *b*_2_ and *b*_3_ paths (the covariances are discussed in the next section). Models with lagged and contemporaneous *b* paths were compared using the BIC and the other model fit indices and informal comparison of the *b* path estimates and their standard errors.

### 2.6 Unmeasured confounding – Lagged and contemporaneous measurement error covariances

One plausible extension of the simplex model is to allow for covariance between measurement errors in the mediator and outcome. Some of this covariance could be thought of as encompassing a type of unmeasured confounding. This could be covariance in errors at the same time point, where factors on a given day are affecting measurement of both mediator and outcome, or covariance between an earlier measure of the mediator and a later measure of the outcome, where there is a more persistent process in play. Such paths can be allowed for when repeated measurements are available, as in Figure 3(c) and (d), which show examples of models with contemporaneous and lagged covariances, respectively. Models with different types of covariances were compared as described in the previous section on time lag of *b* paths.

### 2.7 Precision of the b paths in longitudinal mediation models

To potentially increase the precision of the *b* paths and also in the interest of model parsimony, evidence for setting the measurement error covariances to be equal over time and for setting the *b*_1_, *b*_2_ and *b*_3_ paths to be equal over time was examined. This was done using Wald chi-square tests for model constraints with the degrees of freedom equal to the number of parameter constraints tested. For the covariances, this was initially a test of equality of four contemporaneous covariances or two post-baseline lagged covariances. If this test indicated a significant difference, pairwise tests were done to characterise the differences. For the *b* paths, the Wald test was structured as a test of equality of either three contemporaneous post-randomisation paths (*b*_1_, *b*_2_ and *b*_3_) or two lagged post-randomisation paths (*b*_2_ and *b*_3_).

One other assumption was explored. Theoretically there may be no need for treatment to mediator (*a*_3_ path) and treatment to outcome (*c*_3_ path) paths beyond the end of treatment. The need for these paths was assessed using a Wald chi-square test for these two paths being equal to zero.

The assumptions were tested in the models in the order: equal measurement error covariances, need for *a*_3_ and *c*_3_ paths and equality of *b* paths. If an assumption could be made, it was incorporated in models going forward.

### 2.8 Calculation of indirect effects in longitudinal mediation models

When we extend mediation models to the longitudinal case there are more indirect or mediated paths than the single effect in the simple mediation model. In the simplex models we have fitted using the SEM framework, the mediated effects are straightforward to calculate using the path tracing rules described previously.^8–10,21^ When assuming multivariate normality and linear relationships between variables,^11,12^ this entails finding each path between two variables, multiplying together all parameter estimates on each path and then summing these products together. Cole and Maxwell point out the key issue of choosing the effects of interest in models with several measures of outcome over time . They suggest these would most likely be effects on the final outcome time point, given that data have been gathered to that point for a clinical and/or theoretical reason.^21^ They describe ‘time-specific indirect effects’, which refer to the effects for a given time point, and ‘overall indirect effects’ which are the effects on the final measurement. Indirect effects are those going through any measure of the mediator, with direct effects being those that do not pass through any measure of the mediator.

The main indirect/mediated effect of interest in the PACE example here is the overall indirect effect for the 52 week time point. In order to obtain this effect, all of the time-specific indirect effects would be calculated for this time point and summed together. An example of one of the time-specific effects that would contribute to the overall indirect effect at 52 weeks in the model in Figure 3(a) would be R->M_12_->M_24_->M_52_->Y_52_ (calculated as *a*_1_ × *m*_2_ × *m*_3_ × *b*_3_). An example of a direct or non-mediated effect (does not pass through any measure of the mediator) would be R->Y_12_->Y_24_->Y_52_ (calculated as *c*_1_ × *y*_2_ × *y*_3_). So, the total effect of the randomised treatment R on the final measure of Y_52_, including all indirect and direct effects, would be:

(4) $$Y_{52}=(a_{1}R\cdot m_{2}M_{12}\cdot m_{3}M_{24}\cdot b_{3}M_{52})+$$

(5) $$(a_{1}R\cdot m_{2}M_{12}\cdot b_{2}M_{24}\cdot y_{3}Y_{24})+$$

(6) $$(a_{1}R\cdot b_{1}M_{12}\cdot y_{2}Y_{12}\cdot y_{3}Y_{24})+$$

(7) $$(a_{2}R\cdot m_{3}M_{24}\cdot b_{3}M_{52})+$$

(8) $$(a_{2}R\cdot b_{2}M_{24}\cdot y_{3}Y_{24})+$$

(9) $$(a_{3}R\cdot b_{3}M_{52})+$$

(10) $$(c_{1}R\cdot y_{2}Y_{12}\cdot y_{3}Y_{24})+$$

(11) $$(c_{2}R\cdot y_{3}Y_{24})+$$

(12) $$(c_{3}R)$$

Expressions (4) through (9) are time-specific indirect effects, which summed together, constitute the overall indirect effect (because here we are looking at the final outcome time point). Expressions (10), (11) and (12) are time-specific direct effects (for the 52 week time point), which summed together, constitute the overall direct effect. The total effect can be obtained by summing all effects, or by summing the overall indirect and direct effects. These effects can be easily extended to the simplex models by replacing the observed variables by their latent true score counterparts. The target parameters are the same in the simplex and autoregressive models, but the estimates differ; in the case of the simplex model the parameters incorporate a model-based correction for measurement error. The indirect and direct effects can be assumed to be causal if there is no residual unmeasured confounding or measurement error, no ‘intermediate confounders’, the linearity and multivariate normality assumptions are met and the models are otherwise properly specified. Confidence intervals (CI) for the indirect and direct effects were calculated using 1000 repetitions of the bias-corrected bootstrap.^45–47^ While we focus here on making these assumptions and taking the SEM approach, causal estimators could be elucidated using potential outcomes, following on from the multiple causally ordered mediator estimands put forward by Daniel et al.^45^

### 2.9 Simulation study – the consequences of ignoring measurement error and unmeasured confounding

Some model assumptions were addressed using simulations. Data were simulated and analysed using the Mplus MONTECARLO command. Data were generated under the following three models:

1. Simplex model without measurement error or unmeasured confounding (error covariances) to approximate the autoregressive models (as in Figure 6(c) with ɛ = 1 × 10^−10^).
2. Simplex model allowing for measurement error, no unmeasured confounding (as in Figure 6c).
3. Simplex model allowing for measurement error and unmeasured confounding (as in Figure 6e).

The data generated were then analysed under each of the three types of model in turn. The analyses with equivalent data generation and analysis models served as controls. The true parameter values were obtained by fitting the model of interest to the PACE data, saving the parameter estimates and using them as the true estimates in the MONTECARLO program. The simulation study was based on the two group CBT versus APT comparison, with datasets of n = 50, 100, 320 (the number in two groups in the PACE trial), 640 (the number in four groups in the PACE trial) and 1000 simulated, with 1500 repetitions in each case, as recommended.^48^ Absolute bias was calculated by subtracting the true parameter values from the average parameter estimate across repetitions. Mean square error (MSE) and coverage across repetitions were obtained from the Mplus output.

## 3 Results

**Figure 5.**
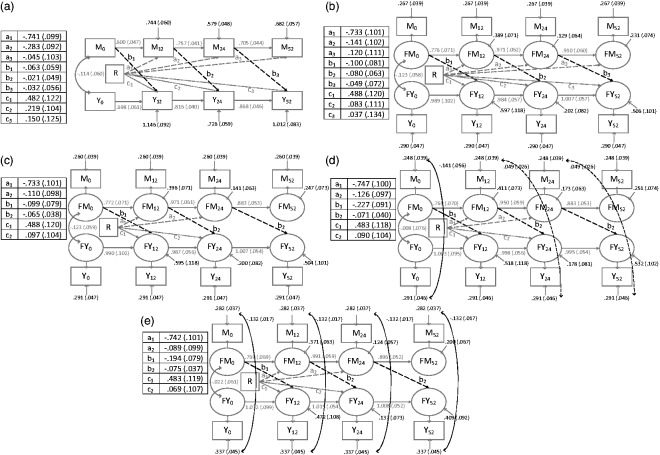
Longitudinal mediator–outcome process models with lagged *b* paths. Numbers are estimate (standard error). R: randomised treatment group; M_0_, M_12_, M_24_, M_52_: mediator measurements taken at baseline, 12 weeks, 24 weeks and 52 weeks post-randomisation; Y_0_, Y_12_, Y_24_, Y_52_: outcome measurements taken at the same time points; FM_0_, FM_12_, FM_24_, FM_52_: true mediator scores at baseline, 12 weeks, 24 weeks, 52 weeks post-randomisation; FY_0_, FY_12_, FY_24_, FY_52_: true outcome scores at the same time points. Paths between observed and latent variables for both the mediator and the outcome process all have factor loadings = 1.

The model diagrams are shown in Figures 5 (lagged *b* paths) and 6 (contemporaneous *b* paths).

**Figure 6.**
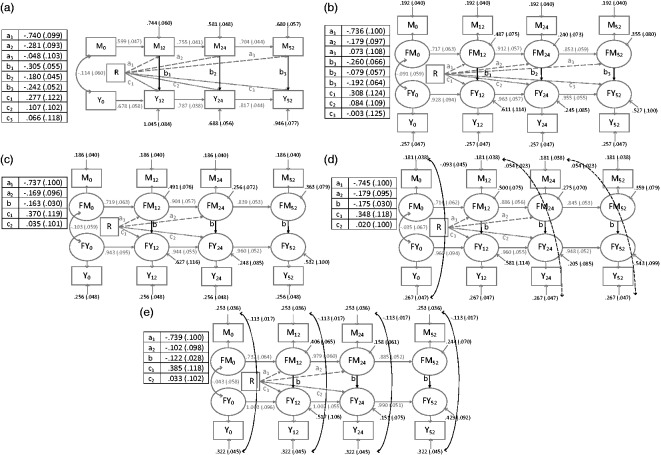
Longitudinal mediator–outcome process models with contemporaneous *b* paths. Numbers are estimate (standard error). R: randomised treatment group; M_0_, M_12_, M_24_, M_52_: mediator measurements taken at baseline, 12 weeks, 24 weeks and 52 weeks post-randomisation; Y_0_, Y_12_, Y_24_, Y_52_: outcome measurements taken at the same time points; FM_0_, FM_12_, FM_24_, FM_52_: true mediator scores at baseline, 12 weeks, 24 weeks, 52 weeks post-randomisation; FY_0_, FY_12_, FY_24_, FY_52_: true outcome scores at the same time points. Paths between observed and latent variables for both the mediator and the outcome process all have factor loadings = 1.

### 3.1 Measurement error

**Figure 7.**
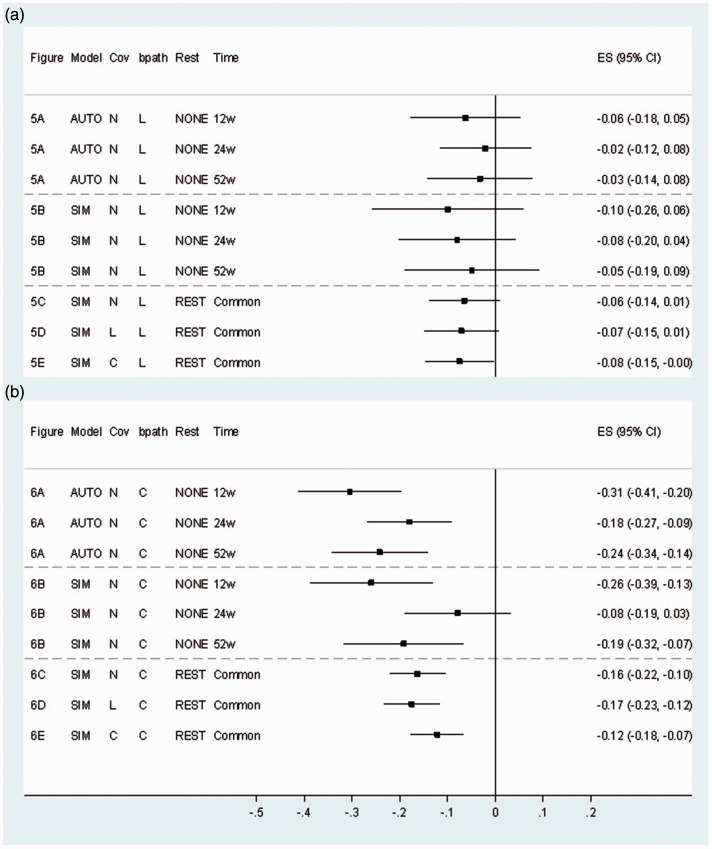
*b* paths (95% CI) for autoregressive and simplex models. (a) Lagged *b* paths; (b) Contemporaneous *b* paths. Cov: covariance type; Rest: restrictions; AUTO: autoregressive model; SIM: simplex model; N: no error covariances; L: lagged; C: contemporaneous; NONE: treatment to 52 week paths present, mediation *b* paths not set equal; REST: treatment to 52 week paths = 0, mediation *b* paths set equal; w: weeks post-randomisation; Common: common *b* path for all post-randomisation time points; ES: estimate; CI: confidence interval.

Model fit indices show that the simplex models fitted better than the comparable autoregressive models (Table 1), suggesting it was important to allow for measurement error. For example, for the models with contemporaneous *b* paths, the simplex model BIC minus the autoregressive model BIC was −24 (comparing Figure 6a and b). For the models with lagged *b* paths, accounting for measurement error had the expected disattenuation effect, i.e. the simplex model *b* paths were larger in magnitude (in Figure 5b versus Figure 5a and Supplementary Table A). However for models with contemporaneous *b* paths, the opposite was true (Figure 6b versus Figure 6a and Supplementary Table A). The effect of taking measurement error into account was also seen in the magnitude of paths between the previous measure of the mediator/outcome and the subsequent measure (i.e. the *m* and *y* paths as shown in Figure 3c). These paths were of larger magnitude in the simplex models (for example, compare Figure 5a and b). Accounting for measurement error led to a small loss in precision (e.g. comparing the *b* paths from autoregressive model Figure 6a to simplex model Figure 6b, also see Figure 7). Evidence suggested that the *a*_3_ and *c*′_3_ paths to the final outcome time point could be set equal to zero in the simplex models (p > 0.43 for all covariance/b path lag combinations), so they were set equal going forward.

**Table 1.** Comparison of fit statistics across autoregressive and simplex models.

General type	Figure #	Covariance type	*b* path type	Model χ^2^	RMSEA (90% CI)	AIC	BIC
Models without parameter restrictions^a^							
Autoregressive	5A	N	L	187.943 df = 20 p < 0.001	0.162 (0.141, 0.184) p < 0.001	6633.276	6753.863
Simplex	5B	N	L	115.696 df = 18 p < 0.001	0.130 (0.108, 0.153) p < 0.001	6565.030	6693.153
Autoregressive	6A	N	C	123.004 df = 20 p < 0.001	0.127 (0.106, 0.149) p < 0.001	6568.337	6688.924
Simplex	6B	N	C	87.706 df = 18 p < 0.001	0.110 (0.088, 0.134) p < 0.001	6537.039	6665.162
Models with parameter restrictions^a^							
Simplex	5C	N	L	117.023 df = 21 p < 0.001	0.120 (0.099, 0.141) p < 0.001	6560.356	6677.174
Simplex	6C	N	C	91.916 df = 22 p < 0.001	0.100 (0.079, 0.121) p < 0.001	6533.249	6646.299
Simplex	5D	L	L	106.322 df = 19 p < 0.001	0.120 (0.098, 0.143) p < 0.001	6553.655	6678.010
Simplex	5E	C	L	47.051 df = 20 p = 0.001	0.065 (0.041, 0.089) p = 0.14	6492.384	6612.970
Simplex	6D	L	C	82.252 df = 20 p < 0.001	0.099 (0.077, 0.121) p < 0.001	6527.585	6648.171
Simplex	6E	C	C	41.383 df = 21 p = 0.005	0.055 (0.030, 0.080) p = 0.34	6484.717	6601.534

χ ^2^: chi-square model fit statistic; RMSEA: root mean square error of approximation; CI: confidence interval; AIC: Akaike’s Information Criterion; BIC: Bayesian Information Criterion; N: no measurement error covariances; C: contemporaneous; L: lagged; df: degrees of freedom.

a Parameter restrictions are treatment to mediator at 52 weeks and treatment to outcome at 52 weeks = 0 and mediator to outcome *b* paths equal over time. Error variances are set equal for the mediator measures and the outcome measures in the simplex models (all models except 5A and 6A).

### 3.2 Time lags of *b* paths

The BIC suggested that models with contemporaneous *b* paths fitted better than models with lagged paths (Table 1). For example, the BIC for the simplex model with contemporaneous *b* paths minus the BIC for the model with lagged *b* paths was −31 (comparing Figures 5c and 6c). The contemporaneous *b* paths were generally larger in magnitude than the lagged *b* paths (Figure 7 and Supplementary Table A).

### 3.3 Unmeasured confounding

In both the cases of the lagged and contemporaneous error covariances, the overall tests for equality of covariances indicated that not all covariances could be assumed equal (p < 0.004 for lagged and p < 0.030 for contemporaneous). For the lagged covariances, there was no evidence that the 12–24 week and 24–52 week covariances were different (p > 0.77), so these were set equal. For the contemporaneous covariances, the general pattern was that the baseline and 24 week covariances were of a smaller magnitude than those at 12 and 52 (data not shown). When considered from the point of view of unmeasured confounding there would seem little basis for a hypothesis of a constant covariance. However, when considered from a measurement model perspective, a parsimonious model would suggest uniform covariances as plausible. The RMSEA and AIC were very similar in models with and without these covariances set equal, with the BIC being smaller in the models with equal covariances. For example, the model shown in Figure 6(e) with equal contemporaneous covariances had a BIC difference of 7 when compared to a model where the covariances were freed (data not shown). The four covariances were therefore set to be equal in models with contemporaneous covariances going forward. The covariances were generally statistically significant in models where they were set equal (see Figures 5d and e and 6d and e).

The inclusion of contemporaneous error covariances had greater impact than the inclusion of lagged error covariances. For example, in the three models with lagged *b* paths in Figure 5(c) to (e), the BICs for the models with no and with lagged error covariances were very similar (Table 1, Figure 5c compared to Figure 5d), however, the differences in BIC for the model with contemporaneous error covariances and the model with none was −64 (Table 1, Figure 5e compared to Figure 5c). There was a similar pattern for models with contemporaneous *b* paths. The only models with RMSEA values consistent with good model fit were those with contemporaneous error covariances (Table 1). In models with contemporaneous *b* paths, the inclusion of contemporaneous covariances decreased the magnitude of the *b* path somewhat (comparing the models in Figure 6c and e, see also Figure 7).

### 3.4 Precision of the b paths

Tests for equality of *b* paths over time showed that these could be set equal both for lagged paths (for equality of *b*_2_ and *b*_3_ paths, p = 0.92 for model with lagged covariances, p = 0.84 for contemporaneous covariances) and contemporaneous paths (for equality of *b*_1_, *b*_2_ and *b*_3_ paths, p = 0.13 for model with lagged covariances, p = 0.12 for contemporaneous covariances). Making this assumption of equal *b* paths over time gave a large precision gain; for example, comparing Figure 6(b) to (c), the smallest standard error in Figure 6(b) with free b paths was 0.057 and in Figure 6(c) with a common b path was 0.030, giving a 47% relative increase in precision. Assuming equal error covariances over time also increased the precision of the *b* path, but by only a small amount in most cases (data not shown).

### 3.5 Type of covariances versus lag of b paths

In the simplex model it appeared that the type of measurement error covariances was more important than the type of *b* paths. For example, in terms of model fit there was little to choose between the two models having contemporaneous covariances but different types of *b* paths in Figures 5(e) and 6(e) (Table 1). On the other hand, when comparing models with the same type of *b* paths but contemporaneous versus lagged covariances (for example, Figure 5e versus Figure 5d), it is clear that the models with contemporaneous covariances fitted the data much better (Table 1).

### 3.6 Best fitting model

In summary, the most plausible model by RMSEA, AIC and BIC was the simplex with contemporaneous *b* paths and error covariances (Table 1 and Figure 6e). As this model had no treatment to 52 week mediator or outcome paths it assumed no direct effect of treatment on physical functioning at 52 weeks, and that any residual effects of treatment had persisted from the post-treatment time point (24 weeks).

### 3.7 Indirect effects in longitudinal simplex mediation models

**Figure 8.**
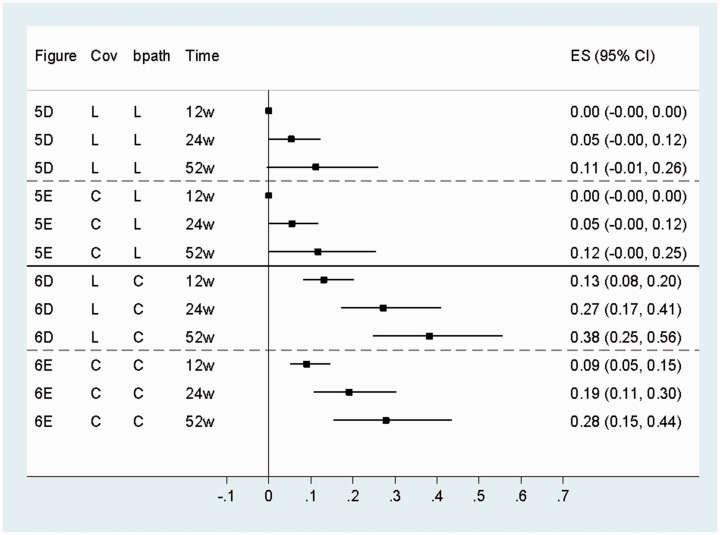
Overall indirect/mediated effects (95% bias-corrected bootstrap CI) for simplex models. Cov: covariance type; L: lagged; C: contemporaneous; w: weeks post-randomisation; ES: estimate; CI: 95% bias-corrected bootstrap confidence interval.

Figure 8 shows the total indirect/mediated effects for each time point, with more detail of the estimates for the various time specific indirect, direct and total and overall effects in Tables B, C and D in the supplementary material. Figure 8 shows the smaller *b* paths in the lagged models led to smaller indirect effects as would be expected. The figure also shows that using path analysis rules to calculate mediated effects from the simplex models gives effects that accumulate over time.

The total effect of treatment group on physical functioning at 52 weeks in the best fitting model with contemporaneous *b* paths and error covariances (Figure 6e) was 0.69 physical functioning SD units (95% CI 0.44, 0.95) (Supplementary Table D). The time specific indirect/mediated effect for this time point indicated that CBT increased physical functioning through the fear avoidance mediator by 0.28 physical functioning SD units (95% bias-corrected bootstrap CI 0.15, 0.44) (Figure 8 and Supplementary Table D). This suggested that approximately 41% of the effect of treatment on physical functioning was mediated through fear avoidance when all repeated measures of both mediator and outcome were modelled and measurement error and unmeasured confounding were taken into account.

The common *b* path assumption that led to greater precision in the *b* paths also led to greater precision in the indirect effects. In the model shown in Figure 6(e), the standard error (SE) for the 52 week indirect effect with this assumption was 0.068, giving a CI of 0.15–0.44 (Figure 8). If the common *b* path assumption was relaxed in this model, the SE was 0.073 and CI 0.18–0.47.

### 3.8 Consequences of ignoring measurement error and unmeasured confounding

**Figure 9.**
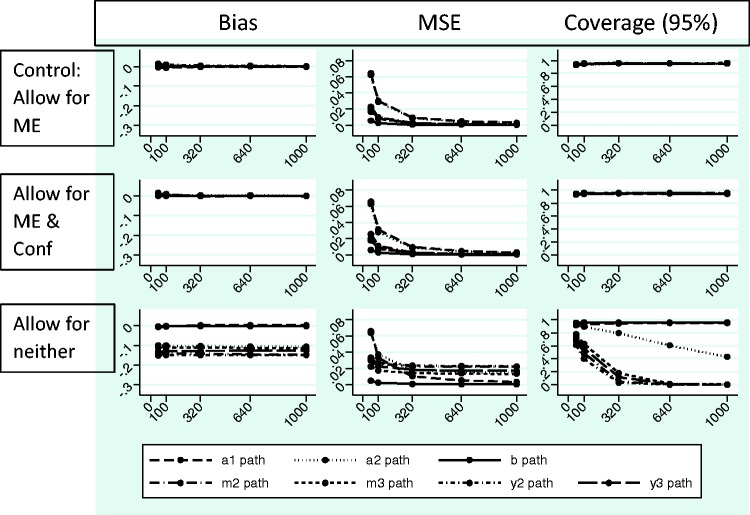
Simulation results – measurement error present. All data generated using simplex models and 1500 repetitions for each sample size, allowing for measurement error (ME), but not for measurement error covariances representing unmeasured confounding (Conf). MSE: mean square error; Control: generated and analysed allowing for ME only when only ME present: Allow for ME & Conf: analysed allowing for both ME and Conf when only ME present; Allow for neither: analysed without allowing for ME or Conf when only ME present.

**Figure 10.**
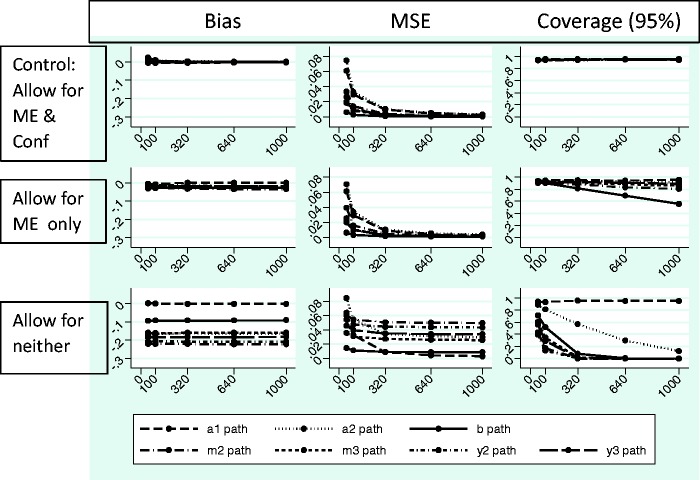
Simulation results – measurement error and confounding present. All data generated using simplex models and 1500 repetitions for each sample size allowing for measurement error (ME) and for measurement error covariances representing unmeasured confounding (Conf). MSE: mean square error; Control: generated and analysed with ME and Conf when both ME & Conf present; Allow for ME only: analyse allowing ME only when both ME & Conf present; Allow for neither: analysed without allowing for ME or Conf when both ME & Conf present.

Figures 9 and 10 show the absolute bias, MSE and coverage results from the simulation study. Each simulation took 25 seconds or less to run using the Mplus program. Figure 9 shows effects of ignoring measurement error when it is present on the *a*_1_, *a*_2_, *b*, *m*_2_, *m*_3_, *y*_2_ and *y*_3_ paths and Figure 10 shows the effect of ignoring measurement error and confounding when they are present on the same paths (the numeric data are available upon request). Models converged across most repetitions in all conditions, with some exceptions when n = 50 (Supplementary Table E). There were problems with the latent variable covariance matrix and the residual covariance matrix for simulations where data were generated and analysed with measurement error, which were alleviated in the case of larger sample sizes. There were also problems with the residual covariance matrix for simulations where data were generated without and analysed allowing for measurement error that did not dissipate with increasing sample size. There was little evidence of effects on bias, MSE and coverage when allowing for measurement error and unmeasured confounding in the case where these were not present (Supplementary Figure A and Figure 9 middle row). However, there were issues with the statistical quantities for most of the paths of interest in the models when measurement error was present but not accounted for (Figures 9 and 10, bottom rows) and to a lesser extent when only unmeasured confounding was ignored (Figure 10, middle row). The effects were somewhat worse when both measurement error and confounding were present and ignored (Figure 10, bottom row) as compared to measurement error alone (Figure 9, bottom row). Effects tended to be worse for the paths connecting the mediator and outcome measures through time, i.e. the *m*_2_, *m*_3_, *y*_2_ and *y*_3_ paths. This is important because these paths contribute to longitudinal mediated effects (see Section 2.8 *Calculation of indirect effects in longitudinal mediation models*).

Except for the MSE, increasing sample size did not generally alleviate problems with the estimates, and where bias did not improve, coverage worsened. It was of note that the *b* path suffered from very poor coverage both when measurement error and confounding were ignored and also to a lesser extent when just confounding was ignored.

## 4 Discussion

The aim of this paper was to examine issues affecting longitudinal mediator–outcome relationships, or *b* paths, in a trial of complex treatments for CFS. The paper focused specifically on measurement error in the mediator, time lags, unmeasured confounding and the assumption of a constant mediator–outcome relationship over time. It was clearly important to account for measurement error and unmeasured confounding, otherwise parameter estimates and mediated effects were biased, which could have led to inferences about estimates being flawed. While lagged mediator–outcome paths would be more consistent with a causal effect, models with contemporaneous mediator–outcome *b* paths fitted better. Assuming a constant mediator–outcome *b* path over time was plausible and brought a large gain in precision. Our findings here using longitudinal measures supported our earlier finding using a single measure of both mediator and outcome that fear avoidance mediated the effect of treatment on physical functioning.^32^

The superiority of the simplex over the autoregressive models and the results of the simulation study clearly showed it was important to account for measurement error in the mediator, perhaps more so than accounting for unmeasured confounding. Models with lagged mediator–outcome *b* paths followed the classical measurement error paradigm where error dampens effects and so taking account of it increased the magnitude of these paths. On the other hand, the contemporaneous mediator–outcome *b* paths were smaller in magnitude when measurement error was taken into account, which is not what we would expect for classical measurement error. Complex effects of accounting for measurement error in multi-equation models have been noted previously.^11^ Measurement error was accounted for in this study by using the simplex models, but it is also important to try to do more to address this issue through improved measurement of mediators and outcomes in the first place.

Accounting for measurement error led to a small loss in precision. Instrumental variables analysis (IV) is another method for coping with confounding and measurement error in predictor variables. It has proven difficult to apply IV methods to mediation analysis so far, mainly due to the absence of strong instruments, leading to imprecise mediator–outcome estimates.^4,14^ In our experience, the use of these repeated measures measurement error models as an alternative to IV has led to much smaller losses in precision.

Lagged mediator–outcome relationships, which would be more consistent with the temporal ordering of a causal process such as mediation, were not supported in the PACE data. This could have been due to the apparent almost simultaneous change in mediator and outcome in these data.^31^ However, it could also be because the first measurement of the mediator was taken too late to capture mediator change prior to change in the outcome. The mid-treatment measurements were taken after participants had received approximately seven sessions of therapy, which was an important time point from the point of view of the trial, but may have been late in the process of mediator change. For example, evidence of gains in the first three sessions of brief psychosocial therapy interventions has been demonstrated for depression.^49^ Studies looking at the trajectories of mediator and outcome change in more detail by taking earlier and more frequent measures of the variables, perhaps even at every session of therapy, could clarify optimal timing and number of measurements.

The potential for unmeasured confounding of the mediator–outcome relationship was allowed for in models through covariances between mediator and outcome errors. The best fitting models were those with contemporaneous error covariances, suggesting there were unmeasured confounders of the mediator and outcome variables at the same time point that needed to be taken into account. Lagged covariances would have been more consistent with unmeasured confounding in a typical ‘simple’ mediator model with one measure of the mediator taken earlier acting on a single later measure of the outcome. There was less evidence for this sort of unmeasured confounding in the PACE data, although this may not be the case in other situations. Allowing for unmeasured confounding is desirable given the attention this issue has been given in the literature, with the approach described here providing one option. In practice there is no single best approach and, for example, the approach here could be extended to incorporate existing sensitivity analysis methods^22–24^ to quantify the level of confounding that would alter these longitudinal model conclusions.

The assumption of equal mediator–outcome *b* paths over time led to greater precision in these estimates. The idea that no matter how or when the mediator is changed it will have the same effect on the outcome is a potentially strong and theoretically appealing assumption. This assumption aligns well with a description of mediation used in programme theory and intervention evaluation. These fields have described mediation analysis as evaluating both an ‘action theory’ – the *a* path in Figure 1 where an intervention seeks to change a mediating variable, and a ‘conceptual theory’ – the *b* path in Figure 1, which is the causal relationship between the mediator and outcome.^50^ Describing the *b* path as the ‘conceptual theory’ fits with thinking of this as a stable relationship existing in nature that can be manipulated by the ‘action theory’ or intervention. This implies that the ‘conceptual theory’ relationship exists in the absence of the intervention and should exist at different points in time. The support of both action and conceptual theories provides evidence for mediation. From a statistical point of view, this assumption led to a large increase in mediator–outcome effect precision, which in turn led to more precise mediated effects. When plausible, making this assumption could be important given the often low power to detect mediated effects.^51^

Many of this study’s strengths lay in the use of high quality data stemming from a rigorously conducted trial, as well as the availability of multiple measurements of mediators and outcomes allowing for the fitting of more complex models. It was only possible to allow for unmeasured confounding in these models because of the availability of multiple measurements. Using a single measurement each of mediator and outcome as is generally done in mediation analysis does not allow for fitting of a model with mediator–outcome covariance, as such a model is not identified. It is also more difficult to account for measurement error in these single measure models, although it can be done if the reliability of the measure is known. At least three measurements and assumptions such as those applied here are needed for identification of all parameters in single-process simplex models. Clinical trials often take only baseline, post-treatment and follow-up measurements, but the mid-treatment measures taken in PACE made it possible to allow for more paths and to explore assumptions in models. Also, given the apparent simultaneous early change in mediators and outcomes in PACE^32^ it may be fruitful to collect more measurements earlier in the process to clarify mediator and outcome trajectories. Having additional repeated measures of mediator and outcome and/or different measurements of the mediator and outcome at each time point could allow for the exploration of further model assumptions. Furthermore, other strengths of this study derive from the methods used. Much of the causal mediation literature has focused on the issue of unmeasured confounding, however, both the simulation results in this study and previous findings^14^ suggest that in the area of mental health measurement error may be of even greater concern than unmeasured confounding. The approach taken here simultaneously addressed both measurement error and some sources of unmeasured confounding induced by correlated measurement error, while avoiding the need to estimate complex sensitivity parameters. As such, this approach provides for another type of sensitivity analysis. In addition, it is likely much easier to gain information about the reliability of measurement, such as we have done here using repeated measures, than it is in most situations to identify and measure all important confounders. This being said, we do not see the approach taken here and the sensitivity analyses described in the literature^15,16,22–24^ as mutually exclusive. For example, the approaches using SEM to study sensitivity to unmeasured confounding^22–24^ could be incorporated into the sorts of measurement models that we have fitted here.

Although there has been some criticism of the use of SEMs to model causal processes, these models have made a large contribution to the modern study of mediation and have been shown here and by others to be a very useful tool for this purpose.^52^ This study showed that modelling of repeated mediator and outcome measurements using SEMs provided flexibility and allowed for exploration of some important assumptions.

There are some limitations of the study. One is that we did not adjust for other potential baseline confounders of the mediator and outcome besides the baseline measures of mediator and outcome. We included a larger list of measured confounders in the initial PACE mediation analysis and so are confident that the relationship between fear avoidance and physical function is robust to confounding.^32^ We would generally advise the inclusion of measured confounders, which requires consideration at the trial design stage. However, the baseline measures of the mediator and outcome are likely the most important confounders^14^ and may also act as proxies for other confounders that have not been included. The longitudinal models used here included baseline and other measures of the mediator and outcome over time, which are also likely to be important confounders. Given this and the results of the initial mediation analysis,^32^ it seems unlikely the inclusion of other variables would have had a large effect on the mediation estimates. In another analysis (not shown), the variable with the largest effect on the relationship between the fear avoidance mediator and physical functioning outcome was baseline fear avoidance, which was included in the models presented here, with baseline work and social adjustment having the second largest effect.^4^ The latter variable changed the mediated effect by less than 0.06 units on the standardised scale. This suggests it is unlikely that including work and social adjustment and other weaker confounders would negate the mediation effects found here. It is possible that measurement error covariances could be less important when more measured confounders are included, and it would be important to explore this. A further consideration was the focus of this study on a subset of PACE data. However, these and other longitudinal models have been used to study the full PACE trial data set, which will be described in another paper. Finally, we have assumed that missing data are missing at random, an assumption that may not have been met. However, the autoregressive and simplex models likely made this assumption more plausible as earlier measures of the mediator and outcome were predictors of later measures in the models. Future research could evaluate the effect of violation of this assumption on estimates of mediated effects.

The simplex models offer advantages over the autoregressive models, including acknowledging measurement error. However, simplex models make some restrictive assumptions, such as assuming effects between different true scores follow a first order autoregressive structure.^42,43^ These may or may not have been plausible assumptions for the PACE data. In addition, the accumulation of mediated effects over time implied by these models may not have best reflected the nature of changes over time in PACE, where at least on average there was greater change in the mediator and outcome variables up to 12 weeks with a plateau afterwards.^31^ These issues may have been reflected in the model fit indices, which suggested that the best model shown here could still be improved upon. We will explore other model types allowing for different assumptions in another paper.

In conclusion, longitudinal SEMs can account for important sources of potential bias in mediation analysis, such as measurement error in the mediator and unmeasured confounding. Optimal application of these methods requires the availability of repeated measures, necessitating consideration at the study design stage. Assuming that no matter how or when the mediator is changed it has the same effect on the outcome was reasonable and gained efficiency. Later phase clinical trials of treatments should aim to address mediation as well as effectiveness hypotheses, keeping in mind these analyses would be enhanced by the inclusion of more and earlier measurements of mediators and outcomes.

## Supplementary Material

Supplementary material
