# Supplementary material for: Measurement error, time lag, unmeasured confounding: Considerations for longitudinal estimation of the effect of a mediator in randomised clinical trials
Source: Stat Methods Med Res. 2016 Sep 19;27(6):1615–33. doi: 10.1177/0962280216666111 (PMC5958412; doi:10.1177/0962280216666111)
Supplement: Supplementary material [file Supplemental_Material.pdf]

Supplementary Material

Table A. Comparison of *b* path estimates (SE) across autoregressive and simplex models

| Figure # | Cov type | *b* path type | BL -> 12 weeks | *b* path 12 -> 24 weeks | *b* path 24 -> 52 weeks | *b* path 12 -> 12 weeks | *b* path 24 -> 24 weeks | *b* path 52 -> 52 weeks |
|---|---|---|---|---|---|---|---|---|
| **Autoregressive** | | | | | | | | |
| Figure 5A | N | L | -0.063 (0.059) | -0.021 (0.049) | -0.032 (0.056) | --- | --- | --- |
| **Simplex** | | | | | | | | |
| Figure 5B | N | L | -0.100 (0.081) | -0.080 (0.063) | -0.049 (0.072) | --- | --- | --- |
| **Autoregressive** | | | | | | | | |
| Figure 6A | N | C | --- | --- | --- | -0.305[a] (0.055) | -0.180[a] (0.045) | -0.242[a] (0.052) |
| **Simplex** | | | | | | | | |
| Figure 6B | N | C | --- | --- | --- | -0.260[a] (0.066) | -0.079 (0.057) | -0.192[a] (0.064) |
| **Simplex with restrictions** | | | | | | | | |
| Figure 5C | N | L | -0.099 (0.079) | -0.065 (0.038) | -0.065 (0.038) | --- | --- | --- |
| Figure 6C | N | C | --- | --- | --- | -0.163[a] (0.030) | -0.163[a] (0.030) | -0.163[a] (0.030) |
| Figure 5D | L | L | -0.227[a] (0.091) | -0.071 (0.040) | -0.071 (0.040) | --- | --- | --- |
| Figure 5E | C | L | -0.194[a] (0.079) | -0.075[a] (0.037) | -0.075[a] (0.037) | --- | --- | --- |
| Figure 6D | L | C | --- | --- | --- | -0.175[a] (0.030) | -0.175[a] (0.030) | -0.175[a] (0.030) |
| Figure 6E | C | C | --- | --- | --- | -0.122[a] (0.028) | -0.122[a] (0.028) | -0.122[a] (0.028) |

Cov = covariance, SE = standard error, BL = baseline, N = no post-baseline covariances, L = lagged, C = contemporaneous

[a]estimate significant at $p \leq 0.05$.

Table B. Time-specific indirect, direct and total effects at 12 weeks (95% bias-corrected bootstrap CI) in fear avoidance/physical functioning simplex models with restrictions

| | **Figure 5D** | **Figure 5E** | **Figure 6D** | **Figure 6E** |
|---|---|---|---|---|
| **Covariance type** | **L** | **C** | **L** | **C** |
| ***b* path type** | **L** | **L** | **C** | **C** |
| **Indirect effects:** | | | | |
| trt -> fm12 -> fy12 | | | 0.130 (0.082, 0.203) | 0.090 (0.051, 0.146) |
| **Total 12 week indirect effect** | | | 0.130 (0.082, 0.203) | 0.090 (0.051, 0.146) |
| **Direct effects:** | | | | |
| trt -> fy12 | 0.483 (0.229, 0.749) | 0.483 (0.231, 0.745) | 0.348 (0.117, 0.607) | 0.385 (0.152, 0.628) |
| **Total 12 week direct effect** | 0.483 (0.229, 0.749) | 0.483 (0.231, 0.745) | 0.348 (0.117, 0.607) | 0.385 (0.152, 0.628) |
| **Total 12 week effect** | 0.483 (0.229, 0.749) | 0.483 (0.231, 0.745) | 0.478 (0.219, 0.735) | 0.475 (0.223, 0.725) |

L = lagged, C = contemporaneous, fm12 = latent true mediator score at 12 weeks, fy12 = latent true outcome score at 12 weeks

Table C. Time-specific indirect, direct and total effects at 24 weeks (95% bias-corrected bootstrap CI) in fear avoidance/physical functioning simplex models with restrictions

| | Figure 5D | Figure 5E | Figure 6D | Figure 6E |
|---|---|---|---|---|
| **Covariance type** | **L** | **C** | **L** | **C** |
| ***b* path type** | **L** | **L** | **C** | **C** |
| **Indirect effects:** | | | | |
| trt -> fm12 -> fy24 | 0.053 (-0.002, 0.123) | 0.055 (-0.002, 0.118) | | |
| trt -> fm12 -> fy12 -> fy24 | | | 0.125 (0.080, 0.184) | 0.090 (0.050, 0.142) |
| trt -> fm12 -> fm24 -> fy24 | | | 0.115 (0.073, 0.177) | 0.088 (0.047, 0.141) |
| trt -> fm24 -> fy24 | | | 0.031 (-0.001, 0.077) | 0.012 (-0.009, 0.045) |
| **Total 24 week indirect effect** | 0.053 (-0.002, 0.123) | 0.055 (-0.002, 0.118) | 0.272 (0.171, 0.410) | 0.191 (0.106, 0.304) |
| **Direct effects:** | | | | |
| trt -> fy12 -> fy24 | 0.482 (0.232, 0.755) | 0.490 (0.236, 0.772) | 0.334 (0.110, 0.604) | 0.385 (0.159, 0.664) |
| trt -> fy24 | 0.090 (-0.119, 0.282) | 0.069 (-0.150, 0.270) | 0.020 (-0.186, 0.213) | 0.033 (-0.179, 0.229) |
| **Total 24 week direct effect** | 0.572 (0.308, 0.831) | 0.560 (0.298, 0.828) | 0.354 (0.100, 0.612) | 0.419 (0.170, 0.663) |
| **Total 24 week effect** | 0.625 (0.385, 0.882) | 0.615 (0.364, 0.867) | 0.625 (0.385, 0.880) | 0.610 (0.365, 0.862) |

L = lagged, C = contemporaneous, fm12 = latent true mediator score at 12 weeks, fm24 = latent true mediator score at 24 weeks, fy12 = latent true outcome score at 12 weeks, fy24 = latent true outcome score at 24 weeks

Table D. Time-specific indirect, direct and total effects at 52 weeks (95% bias-corrected bootstrap CI) in fear avoidance/physical functioning simplex models with restrictions

| | **Figure 5D** | **Figure 5E** | **Figure 6D** | **Figure 6E** |
|---|---|---|---|---|
| **Covariance type** | **L** | **C** | **L** | **C** |
| ***b* path type** | **L** | **L** | **C** | **C** |
| **Indirect effects:** | | | | |
| trt -> fm12 -> fy24 -> fy52 | 0.053 (-0.002, 0.121) | 0.056 (-0.001, 0.117) | | |
| trt -> fm12 -> fm24 -> fy52 | 0.050 (-0.002, 0.127) | 0.055 (-0.0002, 0.128) | | |
| trt -> fm24 -> fy52 | 0.009 (-0.002, 0.036) | 0.007 (-0.005, 0.034) | | |
| trt -> fm12 -> fy12-> fy24 -> fy52 | | | 0.119 (0.079, 0.171) | 0.089 (0.051, 0.137) |
| trt -> fm12 -> fm24-> fy24 -> fy52 | | | 0.109 (0.071, 0.157) | 0.087 (0.048, 0.136) |
| trt -> fm12 -> fm24-> fm52 -> fy52 | | | 0.098 (0.062, 0.149) | 0.078 (0.042, 0.127) |
| trt -> fm24-> fy24 -> fy52 | | | 0.030 (-0.0004, 0.069) | 0.012 (-0.009, 0.044) |
| trt -> fm24-> fm52 -> fy52 | | | 0.026 (-0.001, 0.063) | 0.011 (-0.009, 0.038) |
| **Total 52 week indirect effect** | 0.112 (-0.006, 0.260) | 0.117 (-0.002, 0.255) | 0.382 (0.247, 0.556) | 0.278 (0.154, 0.435) |

| | Figure 5D | Figure 5E | Figure 6D | Figure 6E |
|---|---|---|---|---|
| **Covariance type** | **L** | **C** | **L** | **C** |
| ***b* path type** | **L** | **L** | **C** | **C** |
| **Direct effects:** | | | | |
| trt -> fy12 -> fy24 -> fy52 | 0.479 (0.236, 0.780) | 0.494 (0.239, 0.794) | 0.317 (0.109, 0.582) | 0.382 (0.160, 0.666) |
| trt -> fy24 -> fy52 | 0.090 (-0.118, 0.285) | 0.070 (-0.146, 0.276) | 0.019 (-0.175, 0.201) | 0.033 (-0.171, 0.232) |
| **Total 52 week direct effect** | 0.569 (0.306, 0.841) | 0.564 (0.305, 0.840) | 0.335 (0.092, 0.594) | 0.415 (0.160, 0.655) |
| **Total 52 week effect** | 0.681 (0.432, 0.942) | 0.682 (0.427, 0.944) | 0.717 (0.490, 0.998) | 0.692 (0.441, 0.953) |

L = lagged, C = contemporaneous, fm12 = latent true mediator score at 12 weeks, fm24 = latent true mediator score at 24 weeks, fm52 = latent true mediator score at 52 weeks, fy12 = latent true outcome score at 12 weeks, fy24 = latent true outcome score at 24 weeks, fy52 = latent true outcome score at 52 weeks

Table E. Simulation convergence results

| Generating model | | Analysis model | | Sample size | Convergence | Covariance matrix issues |
|---|---|---|---|---|---|---|
| Measurement error | Unmeasured confounding | Measurement error | Unmeasured confounding | | | |
| N | N | N | N | 50 | 1500/1500 | 0 |
| | | | | 100 | 1500/1500 | 0 |
| | | | | 320 | 1500/1500 | 0 |
| | | | | 640 | 1500/1500 | 0 |
| | | | | 1000 | 1500/1500 | 0 |
| N | N | Y | N | 50 | 1500/1500 | Most |
| | | | | 100 | 1500/1500 | Most |
| | | | | 320 | 1500/1500 | Most |
| | | | | 640 | 1500/1500 | Most |
| | | | | 1000 | 1500/1500 | Most |
| N | N | Y | Y | 50 | 1499/1500 | Most |
| | | | | 100 | 1500/1500 | Most |
| | | | | 320 | 1500/1500 | Most |
| | | | | 640 | 1500/1500 | Most |
| | | | | 1000 | 1500/1500 | Most |
| Y | N | N | N | 50 | 1500/1500 | 0 |
| | | | | 100 | 1500/1500 | 0 |
| | | | | 320 | 1500/1500 | 0 |
| | | | | 640 | 1500/1500 | 0 |
| | | | | 1000 | 1500/1500 | 0 |
| Y | N | Y | N | 50 | 1499/1500 | Most |
| | | | | 100 | 1500/1500 | Many |
| | | | | 320 | 1500/1500 | 1 (0.1%) |
| | | | | 640 | 1500/1500 | 0 |
| | | | | 1000 | 1500/1500 | 0 |
| Y | N | Y | Y | 50 | 1499/1500 | Most |
| | | | | 100 | 1500/1500 | Many |
| | | | | 320 | 1500/1500 | 0 |
| | | | | 640 | 1500/1500 | 0 |
| | | | | 1000 | 1500/1500 | 0 |
| Y | Y | N | N | 50 | 1500/1500 | 0 |
| | | | | 100 | 1500/1500 | 0 |
| | | | | 320 | 1500/1500 | 0 |
| | | | | 640 | 1500/1500 | 0 |
| | | | | 1000 | 1500/1500 | 0 |
| Y | Y | Y | N | 50 | 1496/1500 | Most |
| | | | | 100 | 1500/1500 | Many |
| | | | | 320 | 1500/1500 | 18 (1.2%) |
| | | | | 640 | 1500/1500 | 1 (0.1%) |
| | | | | 1000 | 1500/1500 | 0 |
| Y | Y | Y | Y | 50 | 1498/1500 | Most |
| | | | | 100 | 1500/1500 | Many |
| | | | | 320 | 1500/1500 | 25 (1.7%) |
| | | | | 640 | 1500/1500 | 0 |
| | | | | 1000 | 1500/1500 | 0 |

Figure A. Simulation results – effect of accounting for measurement error and confounding when not present

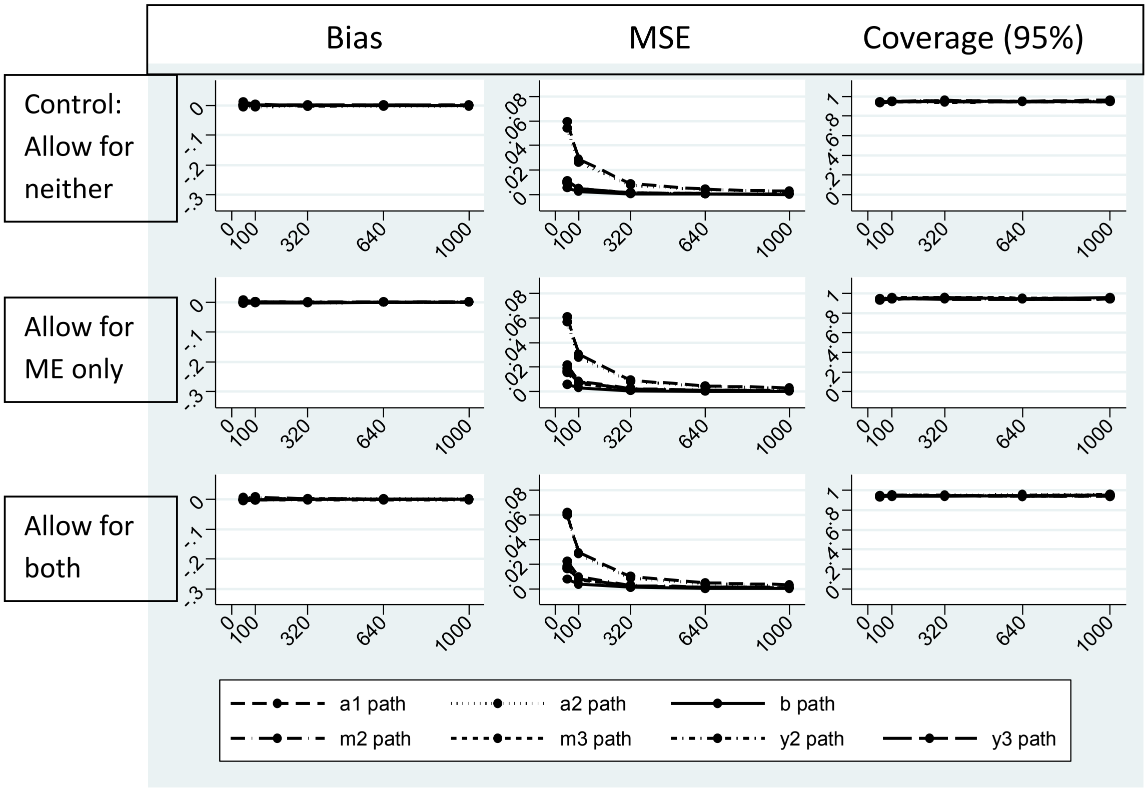


All data generated using simplex models not allowing for measurement error (ME), nor for measurement error covariances representing unmeasured confounding (Conf). MSE = mean square error, Control = generated and analysed without ME and Conf when both ME & Conf absent, Allow for ME only = analyse allowing ME only when both ME & Conf absent, Allow for both = analysed without allowing for ME or Conf when both ME & Conf absent.
